# Chronic intestinal inflammation alters hippocampal neurogenesis

**DOI:** 10.1186/s12974-015-0281-0

**Published:** 2015-04-03

**Authors:** Svetlana Zonis, Robert N Pechnick, Vladimir A Ljubimov, Michael Mahgerefteh, Kolja Wawrowsky, Kathrin S Michelsen, Vera Chesnokova

**Affiliations:** Department of Medicine, Cedars-Sinai Medical Center, Davis Bldg., Room 3019, 8700 Beverly Blvd., Los Angeles, CA 90048 USA; F. Widjaja Foundation Inflammatory Bowel and Immunobiology Research Institute, Cedars-Sinai Medical Center, Los Angeles, CA 90048 USA; Department of Basic Medical Sciences, College of Osteopathic Medicine of the Pacific, Western University of Health Sciences, Pomona, CA 91766 USA

**Keywords:** Inflammatory bowel disease, Chronic peripheral inflammation, Hippocampus, Adult neurogenesis, p21

## Abstract

**Background:**

Adult neurogenesis in the subgranular zone of the hippocampus is involved in learning, memory, and mood control. Decreased hippocampal neurogenesis elicits significant behavioral changes, including cognitive impairment and depression. Inflammatory bowel disease (IBD) is a group of chronic inflammatory conditions of the intestinal tract, and cognitive dysfunction and depression frequently occur in patients suffering from this disorder. We therefore tested the effects of chronic intestinal inflammation on hippocampal neurogenesis.

**Methods:**

The dextran sodium sulfate (DSS) mouse model of IBD was used. Mice were treated with multiple-cycle administration of 3% wt/vol DSS in drinking water on days 1 to 5, 8 to 12, 15 to 19, and 22 to 26. Mice were sacrificed on day 7 (acute phase of inflammation) or day 29 (chronic phase of inflammation) after the beginning of the treatment.

**Results:**

During the acute phase of inflammation, we found increased plasma levels of IL-6 and TNF-α and increased expression of Iba1, a marker of activated microglia, accompanied by induced IL-6 and IL-1β, and the cyclin-dependent kinase inhibitor p21^Cip1^ (p21) in hippocampus. During the chronic phase of inflammation, plasma levels of IL-6 were elevated. In the hippocampus, p21 protein levels were continued to be induced. Furthermore, markers of stem/early progenitor cells, including nestin and brain lipid binding protein (BLBP), and neuronal marker doublecortin (DCX) were all down-regulated, whereas glial fibrillary acidic protein (GFAP), a marker for astroglia, was induced. In addition, the number of proliferating precursors of neuronal lineage assessed by double Ki67 and DCX staining was significantly diminished in the hippocampus of DSS-treated animals, indicating decreased production of new neurons.

**Conclusions:**

We show for the first time that chronic intestinal inflammation alters hippocampal neurogenesis. As p21 arrests early neuronal progenitor proliferation, it is likely that p21 induction during acute phase of inflammation resulted in the reduction of hippocampal neurogenesis observed later, on day 29, after the beginning of DSS treatment. The reduction in hippocampal neurogenesis might underlie the behavioral manifestations that occur in patients with IBD.

## Background

In the adult brain, neurogenesis occurs in the subventricular zone and the subgranular zone (SGZ) of the dentate gyrus (DG) of the hippocampus [1-3]. In humans, new neurons are generated in the hippocampus throughout adulthood [4]. It is well established that neurogenesis is required for many forms of cognitive function involving the hippocampus [5-11]. Low proliferation and differentiation capacity of adult neuronal progenitors correlate with memory dysfunction in humans [12], and in other species, direct or indirect stimulation of neurogenesis can enhance cognitive function [8,13]. Chronic stress decreases hippocampal neurogenesis and results in hippocampal atrophy and depression-like behavior [14,15], and disruption of neurogenesis produces stress-induced depression-like behaviors in mice [10].

During acute or chronic inflammation, circulating pro-inflammatory cytokines can trigger significant and long-lasting behavioral changes, including the development of cognitive impairment and depression [16-19]. Pro-inflammatory cytokines also inhibit adult neurogenesis in the SGZ [20-22]. Therefore, cytokine-induced disruption of neurogenesis might be a key link between chronic inflammation and cognitive impairment and depression.

The cyclin-dependent kinase (Cdk) inhibitor p21^Cip1^ (p21) restrains cell cycle progression and arrests the cell in the G1 phase [23]. Previously, we reported that p21 is induced in early neuronal progenitors and immature neurons in the SGZ, and limits cell proliferation, effectively suppressing neurogenesis [24-26]. Furthermore, acute systemic inflammation and pro-inflammatory cytokines increase SGZ p21 expression, which, in turn, restrains proliferation of hippocampal precursors of neuronal lineage [25].

Unlike acute inflammation, inflammatory bowel disease (IBD) which comprises Crohn’s disease and ulcerative colitis is a chronic condition with a relapsing course and is thought to arise as a result of genetic susceptibility, abnormal intestinal permeability, and abnormal innate and adaptive immune responses to the intestinal microbiota [27,28]. IBD affects about 1.4 million people in the USA and 2.2 million people in Europe. Recent studies demonstrated the connection between intestinal inflammation and changes in brain function. Inflammation in the bowel is associated with a more excitable CNS, as revealed by an increase in seizure susceptibility that correlates with the severity of inflammation. Electrophysiological recordings of hippocampal slices from animals with intestinal inflammation show increased excitability, likely due to increased TNF-α signaling and microglial activation within the brain [29]. Others have demonstrated that chronic experimental colitis increases anxiety behavior in mice [30]. It is likely that peripheral inflammation could account for at least some of the neurological and behavioral symptoms associated with chronic inflammatory diseases. Indeed, IBD patients have higher rates of panic disorder and obsessive-compulsive disorder [31-33] and a twofold increase in the rates of anxiety and depression [31-34]. In addition, people with IBD show cognitive impairment [35-37]. Mechanisms underlying a link between chronic intestinal inflammation and behavior changes are largely unknown.

One well-characterized mouse model of IBD involves repeated administration of dextran sodium sulfate (DSS) in drinking water [38]. DSS is a large molecule (molecular weight (MW) 30,000 to 50,000) that does not cross the blood-brain barrier. Epithelial cell toxicity, increased intestinal permeability, and macrophage activation have been implicated in the deleterious effects of DSS. The DSS model is characterized by colonic epithelial cell lesions and acute (7 to 14 days after the beginning of the treatment) and later chronic intestinal inflammation with neutrophils and macrophages present within damaged segments [39]. After stopping DSS administration, the colon regenerates slowly over several weeks. Typically, mice lose approximately 10% to 15% of body weight during the first two cycles of DSS treatment, but the weight is gradually restored by day 28.

The purpose of this study was to use this mouse model of IBD to examine the effects of chronic peripheral inflammation on new neuron proliferation and development in the hippocampus. We show here that inflammation increases pro-inflammatory cytokine expression in the periphery and in the hippocampus, which, in turn, activates SGZ p21. p21 restrains neuronal progenitor proliferation, and decreased levels of doublecortin (DCX) were observed in DSS-treated mice, indicating reduced hippocampal neurogenesis, whereas glial fibrillary acidic protein (GFAP), a marker of astroglia, was induced. Thus, chronic peripheral inflammation provokes changes in hippocampal neurogenesis that might underlie behavior sequelae of IBD or other chronic peripheral inflammatory diseases.

## Methods

### Experimental animals and chronic intestinal inflammation

This study was carried out in strict accordance with the recommendations in the Guide for the Care and Use of Laboratory Animals of the National Institutes of Health. The protocol was approved by the Institutional Animal Care and Use Committee at Cedars-Sinai Medical Center. Two-month-old C57Bl/6 female mice were used for the experiments. We employed females only because males are more sensitive to the disruptive effects of DSS on colon epithelia and can develop severe inflammation and die. Inflammation was induced using multiple-cycle administration of 3% wt/vol DSS (MP Biomedicals, Santa Ana, CA, USA) in drinking water on days 1 to 5, 8 to 12, 15 to 19, and 22 to 26 as previously described [38].

Mice were sacrificed on day 29 after the beginning of DSS administration. Three independent experiments were carried out (six mice/group in each experiment). Both left and right hippocampi from three mice per treatment group were isolated for protein analysis and from another three mice per treatment group for RNA isolation. All samples were run individually. Trunk blood was collected, and circulating cytokines were measured in serum using ELISA (eBioscience, San Diego, CA, USA).

Additional groups of DSS-treated and control mice (five mice/group) were anesthetized and perfused with paraformaldehyde (4% in 0.1 M phosphate buffer, pH 7.4) for immunohistochemistry studies.

In a separate experiment, five mice that underwent one cycle of DSS water (days 1 to 5) were sacrificed 7 days after the beginning of DSS treatment; hippocampi from three mice from each group were collected for protein analysis and from two mice per group for RNA isolation. Trunk blood was also collected for cytokine measurements. This experiment was carried out twice.

### Evaluation of intestinal inflammation

Colon and cecum were harvested from all experimental mice and scored for the presence and severity of colitis. Histology was used to evaluate inflammation, extent, regeneration, crypt damage, and percentage involvement. The histologic scoring for colitis is based on an established scale using inflammatory and epithelial parameters, as previously reported [38,40,41], and was performed by an experienced pathologist who was blind to the experimental treatment. Disease severity was determined in colon and cecum using a combination of microscopic (grade 0, normal; grade 1, mild; grade 2, moderate; grade 3, severe) and histological (grade 0, histological score 0 to 1; grade 1, 2 to 4; grade 2, 5 to 7; grade 3, 8 to 10; grade 4, 11 to 14) scores.

### Quantitative real time PCR

Total RNA was isolated from hippocampi with TRIzol reagent (Thermo Fisher Scientific, Waltham, MA, USA). After DNAse I treatment (TURBO DNA free, Ambion, Austin, TX, USA), cDNA was synthesized from 3 μg of purified RNA by the SuperScript II First-Strand cDNA synthesis system (Thermo Fisher, Waltham, MA, USA) according to manufacturer’s instructions. Quantitative PCR was performed in a 20-μl reaction using IQ SYBR Green Master Mix in a Bio-Rad IQ5 instrument (Bio-Rad Laboratories, Hercules, CA, USA). Specific validated primers for murine IL-1β, IL-6, TNF-α, and p21 were purchased from SuperArray (Qiagen, Germantown, MD, USA). Triplicate PCR reactions yielded a threshold cycle (Ct) average, with coefficient of variance of <0.05%, and were used to determine ΔCt values [ΔCt = Ct of the target gene minus Ct of the housekeeping GAPDH gene]. A comparative threshold cycle (C_T_) method was used for relative gene expression quantification. All experiments included template-free (water) and reverse transcriptase-minus controls to ensure no contamination. Relative quantities of mRNAs in experimental samples were determined, normalized to glyceraldehyde 3-phosphate dehydrogenase (GAPDH), and expressed in arbitrary units as the fold difference from control (control was taken as one).

### Adult neuronal progenitor cell cultures

Cultures were prepared and conducted according to published protocols [3,42]. Two-month-old C57Bl/6 male mice were sacrificed and the hippocampi dissected and dissociated using Papain Dissociation System (Worthington Biochemicals, Lakewood, NJ, USA). Hippocampal neuronal progenitor cells (NPC) were isolated and cultured using Neural Stem Cell Expansion Kit Neurosphere System in serum-free neurobasal A-medium (R&D Systems, Minneapolis, MN, USA) as described [26]. The single-cell suspension was re-suspended in DMEMF-12 medium supplemented with N-2 Plus Media Supplement, 2 mM L-glutamine, 100 U/ml penicillin, 100 μg/ml streptomycin, 10 ng/ml FGF-2, and 20 ng/ml EGF for 7 days; then, the spheres were collected and dispersed to individual cells, plated on polyornithine-covered culture dishes, and after three passages differentiation was induced by growing in Complete NeuroCult NSC Differentiation Medium (StemCell Technologies, Vancouver, British Columbia, Canada) in the absence of FGF-2 and EGF. Under these conditions, NPC lose nestin and sex-determining region Y-box 2 (SOX2) and primarily generate neuroblasts and astroglia [43-45]. Plated on ECL cell attachment matrix coated coverslips (Upstate Biotechnology (Lake Placid, NY, USA), 5 to 10 μg/cm^2^) in 24 well plates were 5 × 10^4^ cells/ml, and they were cultured for 8 days in differentiating conditions in the presence of murine cytokines, 10 ng/ml IL-1β or 50 ng/ml IL-6 or 20 ng/ml TNF-α (all from Biolegend, San Diego, CA, USA), with half the medium changed and fresh cytokine added every other day. A portion of the cells was collected for Western blot, and the other portion was fixed in 4% paraformaldehyde and immunocytochemistry performed to detect neuronal markers.

### Protein isolation and Western blot analysis

Protein was isolated using an immunoprecipitation kit (Roche Diagnostics, Indianapolis, IN, USA) and Western blot analysis was performed as described [25]. Twenty-five micrograms of protein lysate was resolved by SDS-PAGE and electroblotted onto PVDF membrane (EMD Millipore, Billerica, MA, USA). The membrane was blocked by 5% nonfat dry milk in TBST (50 mM Tris-HCl, pH 7.6, 150 mM NaCl, 0.05% Tween 20) and incubated overnight with primary antibodies at 4°C, followed by incubation with corresponding secondary antibodies (Sigma-Aldrich, St. Louis, MO, USA) for 2 h at room temperature. Immunoreactive bands were detected using Bio-Rad Molecular Imager® ChemiDoc™ XRS And Image Lab™ Software (BioRad Laboratories, Hercules, CA, USA). The following antibodies were used: nestin (1:1,000), brain lipid binding protein (BLBP, 1:800), ionized calcium-binding adapter molecule-1 (Iba1, 1:1,000), and Ki-67 (1:1,000), all from Abcam (Cambridge, MA, USA); p21 (1:300, Cell Signaling Technology, Danvers, MA, USA), DCX (1:1,000), and GAPDH (1:200), all from Santa Cruz Biotechnology (Santa Cruz, CA, USA); and glial GFAP (1:1,000, EMD Millipore, Billerica, MA, USA).

### Immunocytochemistry and immunohistochemistry

Fixed cells or Paraffin brain sagittal sections on coverslips were double-labeled with primary antibodies conjugated with Alexa 488 or Alexa 568 fluorescent dyes (1:400, Thermo Fisher, Waltham, MA, USA). The following primary antibodies were used: p21 (1:50, BD Biosciences, San Jose, CA, USA), SOX2 (1:100 or 1:30), and GFAP (1:50) (both EMD Millipore, Billerica, MA, USA); DCX (1:20) and Ki-67 (1:1,000), all from Abcam (Cambridge, MA, USA). DNA (nuclei) was stained with DAPI (Prolong Gold, Thermo Fisher, Waltham, MA, USA). Antigen retrieval was performed in 10 mM sodium citrate.

*In vitro*, immunoreactive cells were determined by immunocytochemistry and counted in five to ten random fields (total number of cells between 1,000 and 5,000 for each antibody).

*In vivo*, five animals/group were analyzed. Ki-67 and DCX staining were analyzed on 5-μm sagittal sections of the left half of the brain from 0.36 to 0.6 mm lateral to the midline [46]. Positive cells in every third section (a total of 30 sections) from each mouse were counted under × 100 objective, and the sum was multiplied by 3 to estimate the total number of Ki-67- or DCX-positive cells in the region. SOX2, p21, and DCX staining were analyzed on 5-μm sagittal sections of the left half of the brain from 0.46 to 0.6 mm lateral to the midline. Six slides were analyzed for each animal. Some sections were double-labeled to detect DCX and Ki-67, p21 and SOX2, or p21 and DCX co-localization. Cells were counted if they were in or touching the SGZ and were excluded if they were more than two cell diameters away from the SGZ [47].

Samples were imaged with a Leica TCS/SP spectral confocal scanner (Leica Microsystems, Mannheim, Germany) in dual emission mode to distinguish autofluorescence from specific staining.

### Statistical analysis

Nonparametric Wilcoxon rank sum test was used to test the differences in protein and mRNA levels across the groups. The average numbers of cells positive for Ki-67, DCX, or for both (Ki-67^+^DCX^+^) were compared using multivariate analysis of variance (MANOVA) followed by the *post hoc* Tukey test. The average numbers of cells per slide positive for SOX2, p21, DCX, or doubly positive (p21^+^ SOX2^+^ and p21^+^ DCX^+^) were compared using the Student *t*-test with Satterthwaite correction.

## Results

### Chronic intestinal inflammation in DSS-treated mice

**Figure 1:**
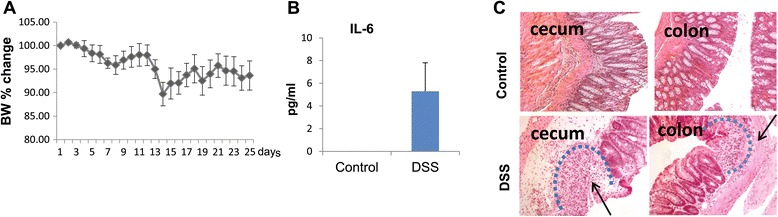
**Chronic intestinal inflammation in mice on day 29 after the beginning of DSS treatment. (A)** Cumulative change of body weight (BW) in the course of dextran sodium sulfate (DSS) treatment from three independent experiments; *n* = 15 mice analyzed. **(B)** Serum IL-6 levels in control and DSS-treated mice (*n* = 8). **(C)** Immunohistochemical analysis of intestinal inflammation (H&E). Inflammation sites are marked by dotted lines. Cecum: Arrow points to the site of severe transmural inflammation with loss of entire crypts and endothelium and infiltration of inflammatory cells. Colon: Arrow points to severe submucosal inflammation with loss of crypts while the surface epithelium is still intact and to infiltration of inflammatory cells.

To induce intestinal inflammation, mice received four cycles of DSS or normal drinking water (controls) for 26 days. Body weight was monitored twice a week. Loss of 7% to 15% body weight initially was observed in DSS-treated animals, and normal weight was restored by day 26 (Figure 1A). The mice were sacrificed on day 29 after the beginning of DSS administration. Trunk blood was collected, and serum levels of TNF-α and IL-6 were measured. TNF-α was below detection in both experimental and control groups of animals. IL-6 was below the level of detection (<2.5 pg/ml) in control mice, whereas it was present in the serum of DSS-treated animals indicating the presence of systemic inflammation (Figure 1B). Signs of intestinal inflammation were assessed histologically by a trained pathologist to evaluate the extent, regeneration, crypt damage, and percentage involvement in cecum and colon as described [38,41,48]. All animals exhibited signs of intestinal inflammation as evidenced by infiltration of inflammatory cells and loss of crypts (microscopic scores 1 to 2, average histological scores 10.2 ± 1.7, *n* = 15) on day 29 (Figure 1C).

### Chronic intestinal inflammation reduces hippocampal neurogenesis

**Figure 2:**
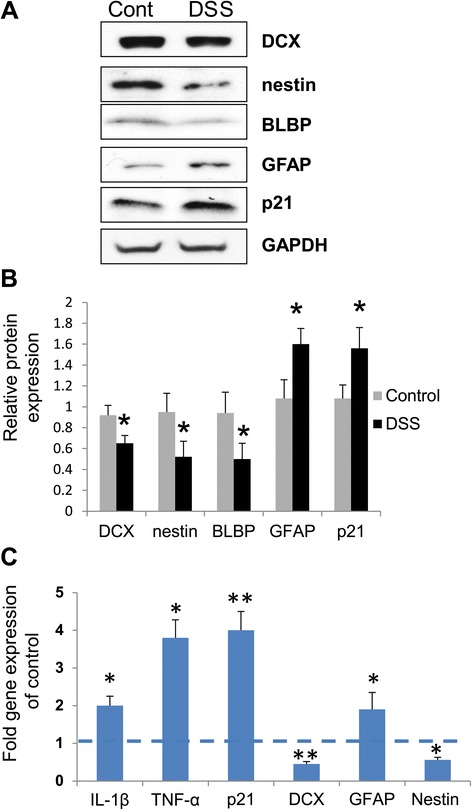
**Chronic intestinal inflammation negatively affects hippocampal neurogenesis.** Mice were sacrificed on day 29 after the beginning of DSS treatment. **(A)** Western blot analysis of markers of neurogenesis and p21. Three independent experiments were performed, and representative blots are shown depicting pooled samples derived from DSS-treated and control mice; **(B)** intensities of protein bands were quantified from nine individual samples run together, normalized to GAPDH and presented as a fold change relative to control animals; **(C)** mRNA levels of proinflammatory cytokines, markers of neurogenesis, and p21 in the hippocampus of DSS-treated mice. Data are shown as mean ± SEM of three independent experiments. All samples from three experiments (*n* = 9) were run together in triplicates and normalized against GAPDH. Results are expressed in fold change *vs*. control taken as 1 (broken line); *p < 0.05, **p < 0.01. BLBP, brain lipid binding protein; Cont, control; DCX, doublecortin; DSS, dextran sodium sulfate; GAPDH, glyceraldehyde 3-phosphate dehydrogenase; GFAP, glial fibrillary acidic protein.

At the conclusion of the experiment on day 29, RNA and protein were isolated from the whole hippocampi. We analyzed mRNA levels of pro-inflammatory cytokines, as well as neuronal markers in the hippocampi of DSS-treated mice. Western blot analysis of the neuronal markers showed decreased levels of nestin and BLBP, both markers of stem/early progenitor cells, whereas levels of GFAP were up-regulated in DSS-treated mice, indicative of activated astroglia. p21 was also induced compared to the controls, and DCX protein levels were decreased, indicating that neurogenesis was reduced in DSS-treated mice (Figure 2A,B). Real-time PCR results indicated that IL-1β and TNF-α mRNA levels were increased more than twofold accompanied by a fourfold increase in p21 mRNA levels, whereas nestin, a marker of stem/early progenitor cells, and DCX, a marker of newly developing neurons, were both down-regulated. At the same time, GFAP mRNA expression was induced (Figure 2C).

**Figure 3:**
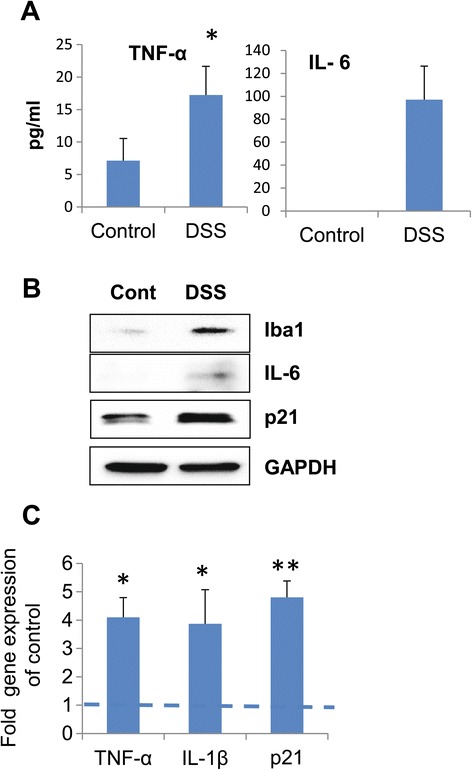
**Acute intestinal inflammation activates hippocampal microglia.** Mice were sacrificed on day 7 after the beginning of DSS treatment. **(A)** Plasma levels of pro-inflammatory cytokines (*n* = 6/group); **(B)** Western blot analysis of Iba1, IL-6, and p21 in the hippocampus (*n* = 3/group). Experiment was repeated two times, and representative blots are shown; **(C)** mRNA levels of pro-inflammatory cytokines and p21. Data are shown as mean ± SEM of two independent experiments. Samples from two experiments (*n* = 4/group) were run together in triplicates and normalized against GAPDH. Results are expressed in fold change *vs*. control taken as 1 (broken line); *p < 0.05; **p < 0.01. Cont, control; DSS, dextran sodium sulfate; GAPDH, glyceraldehyde 3-phosphate dehydrogenase.

The observed changes in neurogenesis could be a result of the action of cytokines released by activated microglia. We therefore tested the expression of Iba1, a marker of activated microglia. Iba1 was not induced in the hippocampi of DSS-treated mice on day 29 at the conclusion of experiment (data not shown). In a separate experiment, we sacrificed a group of mice 7 days after the beginning of treatment (one cycle of DSS), during the acute phase of inflammation. At this time point, plasma levels of TNF-α were elevated. IL-6 was below detection in control mice, whereas was significantly elevated in DSS-treated animals, indicating the presence of systemic inflammation (Figure 3A). These mice exhibited increased Iba1 and IL-6 protein levels, in accordance with induced IL-1β and TNF-α mRNA expression in the hippocampus (Figure 3B,C). High-cytokine levels might trigger p21 as shown previously [25,49]. Indeed, markedly up-regulated p21 mRNA and protein levels were also observed in the hippocampi of mice in the acute phase of inflammation (Figure 3B,C).

### Pro-inflammatory cytokines induce p21 and decrease neurogenesis in NPC

**Figure 4:**
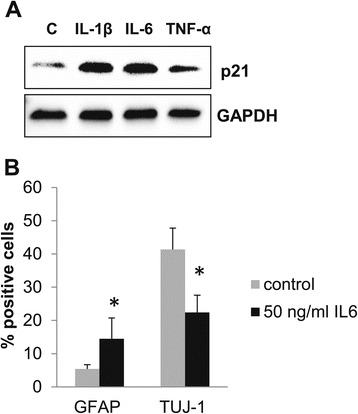
**Proinflammatory cytokines induce p21 and decrease neurogenesis in NPC. (A)** Western blot analysis of NPC differentiated in the presence of 10 ng/ml IL-1β or 50 ng/ml IL-6 or 20 ng/ml TNF-α for 8 days. **(B)** The graph depicts the percentage of Tuj-1^+^ neuroblasts and GFAP^+^ glia cells among NPC cells differentiated untreated (control) or in the presence of 50 ng/ml IL-6. Data are presented as a mean ± SEM, *p < 0.05. GAPDH, glyceraldehyde 3-phosphate dehydrogenase; GFAP, glial fibrillary acidic protein; Tuj-1, neuron-specific class III beta-tubulin.

To further examine mechanisms underlying DSS-induced decrease in neurogenesis, we isolated hippocampal neuronal progenitor cells from naïve mice and allowed these cells to differentiate in the presence of 10 ng/ml IL-1β or 50 ng/ml IL-6 or 20 ng/ml TNF-α for 8 days. Western blot analysis shows that all three cytokines markedly increased p21 expression in differentiating neuronal progenitors (Figure 4A).

We next tested the direct effect of one of the cytokines, IL-6, on NPC differentiation. Cells were double-stained for βIII-tubulin, a marker of committed neuroblasts that is recognized by antibodies to Tuj-1, and for GFAP, and the percent of cells expressing each antigen was calculated. Exposure of NPC to IL-6 resulted in decreased number of Tuj-1-positive cells (from 41% ± 6.4% in untreated cells to 22.39% ± 5.2% in IL-6-treated cells, *p* < 0.05), while the number of GFAP-positive cells increased (from 5.41% ± 1.3% in untreated cells to 14.51% ± 5.3% in IL-6 treated cells, *p* < 0.05) (Figure 4B).

### Chronic intestinal inflammation induces SGZ p21 and suppresses neuronal lineage proliferation

**Figure 5:**
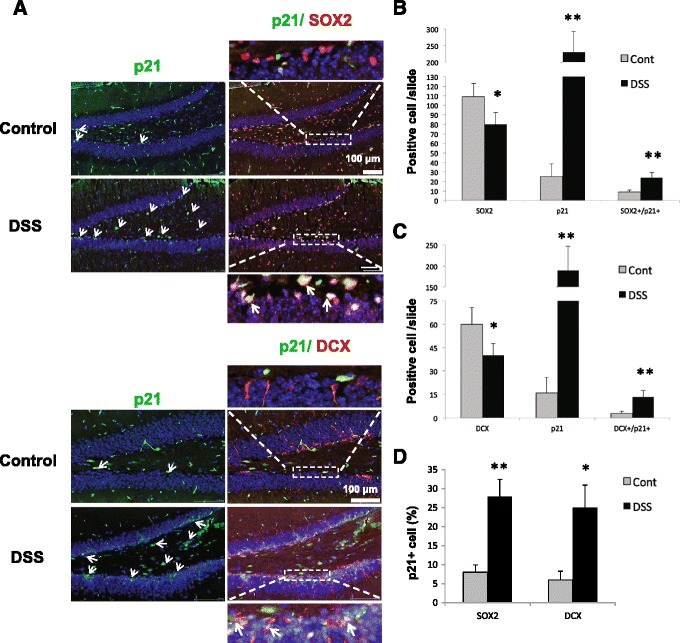
**Chronic intestinal inflammation induces SGZ p21. (A)** The confocal microscopic analysis shows that p21 (nuclear, green) is co-localized with SOX2 (nuclear, pink) or DCX (cytoplasmic, red) and is more abundant in the SGZ of DSS-treated hippocampus. Five slides/group were analyzed and representative images are shown; arrows indicate cell expressing p21 and co-localization of p21 with SOX2 or DCX. **(B)** Average number of SOX2^+^, p21^+^, and SOX2^+^/p21^+^ cells/per slide. **(C)** Average number of DCX^+^, p21^+^, and DCX^+^/p21^+^ per slide. **(D)** Percent of p21^+^ cell in SOX2^+^ or DCX^+^ cells/ per slide in the hippocampus of control and DSS-treated mice. Six slides/mouse/five mice were analyzed. Data are presented as a mean ± SEM. Statistical analysis was performed with the Student *t*-test with Satterthwaite correction. *p < 0.05; **p < 0.01. Cont, control; DCX, doublecortin; DSS, dextran sodium sulfate; SOX2, sex-determining region Y-box 2.

We showed previously that p21 is exclusively expressed in the SGZ of the hippocampus [24,26]. During the chronic phase of inflammation, immunofluorescent staining and confocal analysis of paraffin sections revealed increased number of p21^+^ in the SGZ of DSS-treated mice. We also observe increased number of p21^+^ cells in the hilus. Intense p21 staining was observed in SOX2^+^ and in DCX^+^ cells (Figure 5A). Light green p21^+^ cells negative for SOX2 or DCX likely represent p21^+^nestin^+^ cells as was shown earlier [26]. Average cell number positive for p21, SOX2, and DCX, and doubly positive p21^+^/SOX2^+^ and p21^+^/DCX^+^, was calculated, and an approximately tenfold increase in p21^+^ cells, and an increased percentage of SOX^+^ (8.2% ± 1.9% *vs*. 28% ± 4.5%, *p* < 0.01) and DCX^+^ (6.1% ± 2.3% *vs*. 25% ± 5.9%, *p* < 0.05) cells expressing p21, was detected in DSS-treated animals as compared to controls (Figure 5A,B,C,D), in agreement with our Western blot data (Figure 2B,C). Thus, DSS treatment resulted in the increased number of neuronal progenitors expressing p21.

**Figure 6:**
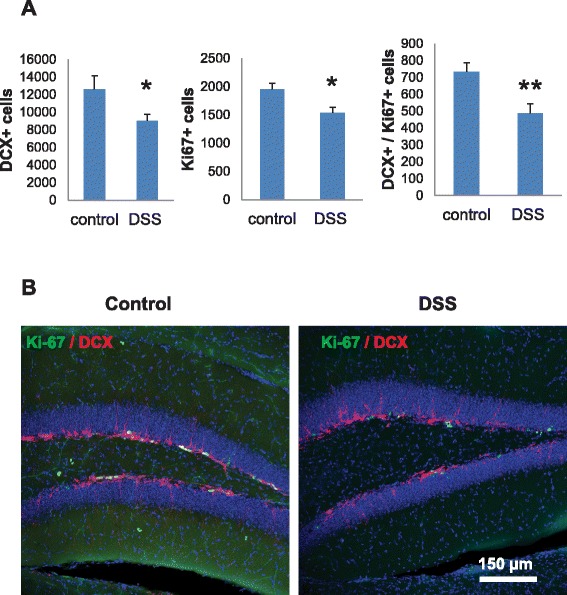
**Chronic intestinal inflammation suppresses proliferation of neuronal lineage. (A)** Numbers of DCX^+^, Ki-67^+^, and DCX^+^/Ki-67^+^ cells in the SGZ of control and DSS-treated mice. For each sample, hippocampi from five mice/group were analyzed. Data are presented as a mean ± SEM. Multivariate analysis of variance (MANOVA) showed significant group effect in the cell count positive for Ki-67^+^, DCX^+^, and Ki-67^+^/DCX^+^ $\left({\mathrm{F}}_8^3=4.33,\ \mathrm{p} = 0.0432\right); \ast \mathrm{p}\le 0.05;\ast \ast \mathrm{p}<0.01;$ **(B)** The confocal microscopic analysis of proliferating DCX^+^/Ki-67^+^ cells in the SGZ of control and DSS-treated mice. DCX cytoplasmic, red; Ki-67 nuclear, green. DCX, doublecortin; DSS, dextran sodium sulfate.

Ki-67 is a marker of cell proliferation. To further assess the effects of chronic inflammation on the proliferation of neuronal progenitors in the hippocampus, paraffin sections were co-labeled with DCX and Ki-67 antibodies. A decreased number of DCX^+^, Ki-67^+^, and DCX^+^/Ki-67^+^ co-labeled cells in the SGZ were observed in the hippocampi of DSS-treated mice (Figure 6A,B), confirming diminished neuronal lineage proliferation. Thus, the decline of neuronal progenitor proliferation in DSS-treated mice was associated with p21 induction in the SGZ of hippocampus.

## Discussion

DSS-induced intestinal inflammation has two phases: acute (days 5 to 14), with pro-inflammatory cytokine release and extensive weight loss, and chronic (days 20 to 28), when the cytokine profile changes to T-helper IL-12/INF-γ secretion, and the weight loss partially or completely recovers [39]. This resembles the course of IBD in humans, characterized by acute disease exacerbation followed by remissions. In our experiments, we have not assessed intestinal inflammation during the acute phase of experiment. However, all experimental animals still exhibited signs of intestinal inflammation on day 29. At that time point, the pathological analysis indicated the presence of inflammation in both the colon and cecum concordant with the increased levels of circulating pro-inflammatory cytokine IL-6 in DSS-treated mice.

Peripheral inflammation could affect hippocampal neurogenesis by several mechanisms. Cytokines in the systemic circulation can access the brain [50,51], or cytokines produced in periphery can signal the brain through the vagal nerve [52]. We as well as others have demonstrated that systemic administration of lipopolysaccharides (LPS) induced the expression of pro-inflammatory cytokines in the brain [25,51,53-55]. Engagement of this immune system-to-brain communication ultimately leads to the activation of resident microglia. Activated microglia can either diminish the production and survival of new neurons or protect developing neurons from apoptosis depending on the duration of inflammation, levels of activation, and the secreted cytokine profile [56-59]. Microglia activation impacts not only the production, but also the migration and the recruitment of new neurons [60]. During the acute phase of colitis, on day 7, DSS treatment stimulated resident hippocampal microglia, evidenced by increased Iba1 protein levels. The effects of activated microglia depend on the factors being released [60,61]. Increased levels of pro-inflammatory cytokines, as observed in hippocampi of DSS-treated mice, can be detrimental to newly developing neurons. Cytokines can be expressed not only by activated microglia, but also by astroglia and neurons [62]. Regardless of the cells of origin, local pro-inflammatory cytokines can negatively affect hippocampal neurogenesis, as was demonstrated for TNF-α, IL-6, and IL-1β [20-22,25,63]. In mice, approximately 3 weeks are required for the new neurons to mature [64,65]; therefore, the effects of activated microglia on day 7 may manifest 3 weeks later. Indeed, neurogenesis was markedly diminished in animals sacrificed on day 29 after DSS treatment.

Although the mechanism by which pro-inflammatory cytokines reduce neurogenesis is not fully understood, we believe that p21 might be a key mediator of this process. p21 can be stimulated directly by pro-inflammatory cytokines [66-68], In addition, p21 can be stimulated by transcription factor Notch1 expressed in neuronal stem cells [69]. Notch1 is known to suppress cell proliferation by stimulating p21 expression [70] and can induce p21 in neuronal progenitors in response to cytokine activation, suppressing neurogenesis and stimulating generation of astrocytes [71]. Although Iba1 protein levels were not increased in the chronic phase of intestinal inflammation, we still observed up-regulation of p21 and GFAP, a marker for astroglia. It is likely that cytokines triggered hippocampal p21 expression in acute as well as in the chronic phase of inflammation. Additional support of this hypothesis comes from our *in vitro* experiments. Exposure of WT NPC with all three pro-inflammatory cytokines resulted in up-regulation of p21. Induced p21 might be also a result of increased levels of circulating cytokines in DSS-treated mice.

We reported previously [26] that p21 is expressed exclusively in the SGZ and is co-localized with SOX2, nestin, and DCX. p21 is also induced by acute systemic inflammation (LPS injection) in neuronal nestin^+^/SOX2^+^ and DCX^+^ progenitors, but not in GFAP^+^ astroglia [25]. We show here that the total number of p21^+^ cells was dramatically increased, and the percentage of SOX2^+^ and DCX^+^ cells expressing p21 was also increased approximately threefold in the hippocampus of DSS-treated mice. Moreover, the total number of DCX^+^ cells, and the number of co-labeled DCX^+^/Ki-67^+^ proliferating cells, was decreased after DSS treatment. It is plausible that in DSS-treated mice, increase in p21 expression might be responsible for the decreased early neuronal progenitor proliferation, evidenced by reduced nestin, BLBP, and DCX levels. Our previous results showing an increased rate of hippocampal neurogenesis in p21^−/−^ mice support this hypothesis [24,25].

p21 induction is associated with increased number of GFAP-positive cells *in vitro*, and *in vivo*, with increased levels of GFAP in the hippocampus in DSS-treated mice. However, the increased GFAP expression observed *in vivo* likely is attributed to activated astroglia, as the impact of newly developing astrocytes would be negligible compared to the total number of astrocyte in this region.

Peripheral inflammation has been suggested as a risk factor for developing mood, psychotic disorder, and cognitive impairment [72] and can also affect hippocampal neurogenesis. Acute administration of LPS results in long-lasting effects on neurogenesis and spatial memory in rodents, induces depression-like behavior and anxiety, and suppresses proliferation and survival of new neurons in the SGZ [72,73]. Moreover, early life inflammatory challenge (for example, LPS injection) produces long-lasting anxiety and depression-like behavior and spatial memory impairment and suppresses neurogenesis [16,74,75]. Our results suggest that chronic intestinal inflammation can also negatively impact proliferation and maturation of neuronal precursors eventually resulting in the reduction of DG granular cell population and can thereby influence the properties and functioning of hippocampal circuits. Given the purported role of hippocampal neurogenesis in cognitive function and depression, its reduction during chronic intestinal inflammation might be the cause of behavioral changes including cognitive symptoms and mood disorders that occur in patients with IBD.

## Conclusions

Chronic intestinal inflammation suppresses hippocampal neurogenesis. Increased levels of pro-inflammatory cytokines systemically and in the hippocampus have detrimental effects on proliferation of progenitors of neuronal lineage. Cytokine-induced p21 might play an important role in restraining neuronal progenitor proliferation. Deficient hippocampal neurogenesis may underlie increased rate of mood disorder and cognitive impairment observed in IBD patients.

## Abbreviations

BLBP: brain lipid binding protein
Cdk: cyclin-dependent kinase
DCX: doublecortin
DG: dentate gyrus
DSS: dextran sodium sulfate
GAPDH: glyceraldehyde 3-phosphate dehydrogenase
GFAP: glial fibrillary acidic protein
Iba1: ionized calcium-binding adapter molecule-1
IBD: inflammatory bowel disease
LPS: lipopolysaccharides
MANOVA: multivariate analysis of variance
MW: molecular weight
NPC: neuronal progenitor cells
SGZ: subgranular zone
SOX2: sex-determining region Y-box 2
